# Can Plant Lectins Help to Elucidate Insect Lectin-Mediated Immune Response?

**DOI:** 10.3390/insects12060497

**Published:** 2021-05-27

**Authors:** Pengyu Chen, Kristof De Schutter, Els J. M. Van Damme, Guy Smagghe

**Affiliations:** 1 Department of Plants and Crops, Faculty of Bioscience Engineering, Ghent University, 9000 Ghent, Belgium; pengyu.chen@ugent.be (P.C.); Kristof.Deschutter@Ugent.be (K.D.S.); 2 Department of Biotechnology, Faculty of Bioscience Engineering, Ghent University, 9000 Ghent, Belgium; ElsJM.VanDamme@UGent.be

**Keywords:** insect lectin, plant lectin, innate immunity, cellular immunity, humoral immunity, C-type lectin

## Abstract

**Simple Summary:**

Lectins are proteins that can recognize and selectively bind specific sugar structures. These proteins are present in all kingdoms of life, including plants, animals, fungi and microorganisms and play a role in a broad range of processes. The interactions between lectins and their target carbohydrates play a primordial role in plant and animal immune systems. Despite being the largest and most diverse taxa on earth, the study of lectins and their functions in insects is lagging behind. To study the role of insect lectins in the immune response, plant lectins could provide an interesting tool. Plant lectins have been well characterized and many of them possess immunomodulatory properties in vertebrate cells. The increasing knowledge on the immunomodulatory effects of plant lectins could complement the missing knowledge on the endogenous insect lectins and contribute to understanding the processes and mechanisms by which lectins participate in insect immunity. This review summarizes existing studies of immune responses stimulated by endogenous or exogenous lectins.

**Abstract:**

Lectins are carbohydrate-binding proteins that recognize and selectively bind to specific sugar structures. This group of proteins is widespread in plants, animals, and microorganisms, and exerts a broad range of functions. Many plant lectins were identified as exogenous stimuli of vertebrate immunity. Despite being the largest and most diverse taxon on earth, the study of lectins and their functions in insects is lagging behind. In insects, research on lectins and their biological importance has mainly focused on the C-type lectin (CTL) family, limiting our global understanding of the function of insect lectins and their role in insect immunity. In contrast, plant lectins have been well characterized and the immunomodulatory effects of several plant lectins have been documented extensively in vertebrates. This information could complement the missing knowledge on endogenous insect lectins and contribute to understanding of the processes and mechanisms by which lectins participate in insect immunity. This review summarizes existing studies of immune responses stimulated by endogenous or exogenous lectins. Understanding how lectins modulate insect immune responses can provide insight which, in turn, can help to elaborate novel ideas applicable for the protection of beneficial insects and the development of novel pest control strategies.

## 1. Introduction

Lectins are unique proteins that are characterized by their ability to selectively bind to specific carbohydrate residues. These sugar structures can be monosaccharides, disaccharides, or polysaccharides, and can be present as free sugars or as glycoconjugates linked to proteins and lipids. In the past, lectins were found to agglutinate red blood cells; therefore, they were often referred to as “hemagglutinins” or “agglutinins” [1]. Subsequent research indicated that agglutination is not universal for all lectins. Only some plant lectins will agglutinate certain types of cells, and this aggregation of cells can be blocked by preincubation with specific sugars. Consequently the word “lectin”, meaning “to select”, was introduced to replace the term hemagglutinin [2].

Because of their selectivity in carbohydrate binding, lectins play crucial roles in a multitude of biological processes in plants, animals, and microorganisms. For example, many plant lectins serve as defense proteins and are harmful to insects or pathogens [3]. Similarly, some animals can secrete lectins that can kill bacteria by forming pore structures on their membranes [4]. Bacteria use their surface lectins to adhere to host cells for invasion [5]. Inside cells, lectins participate in protein quality control [6]. In the extracellular matrix, some lectins alter ion transport [7]. Secreted lectins have also been reported to be involved in host immunity due to their ability in pathogen recognition [8,9].

This review focuses on the role of lectins in insect immunity. In addition, the question is raised whether the study of immunomodulatory effects of plants lectins can complement our knowledge of the functions of insect lectins in the immune response. A better understanding of the lectin-related, insect-immunity-related processes can provide a perspective for the protection of beneficial/economical insects, and can also help in the development of new pest control strategies.

## 2. Insect Innate Immunity

Animals are frequently challenged by invading pathogens such as fungi, bacteria, viruses, parasites, etc. Furthermore, they also harbor a microbiome in tissues such as the intestine and the hemolymph [10]. To maintain homeostasis and system integrity, animal hosts must regulate their own microbiota and eliminate pathogen infection through an elaborate immune system [11]. While mammalians have both an adaptive (depending on memory immune cells) and an innate immunity system, insects mainly depend on innate immunity when threatened by pathogens. Nonetheless, insects have evolved to be very successful organisms, occupying almost every habitat and ecological niche. This is due to a strong innate immune system consisting of a cellular and a humoral component (reviewed by [12,13,14]). The cellular defense is initiated instantly when pathogens are detected and results in the phagocytosis of smaller pathogens or encapsulation of bigger invaders [13,14]. The humoral defense is a relatively slow response and involves the production of a series of antimicrobial peptides (AMPs), complement proteins, lysozymes, protease inhibitors, reactive oxygen species (ROS), and enzyme cascades leading to the formation of melanin and clotting [12,14].

The cellular or humoral immunity system depends on the presence of immune cells of different types. These immune cell types can differ between insect species. For example, the mosquito *Aedes aegypti* has more kinds of immune cells identified than *Drosophila* [15]. The immune cells, called hemocytes due to their presence in the hemolymph, have differentiated from prohemocytes and are mainly composed of three highly differentiated cell types: the plasmatocytes, crystal cells, and lamellocytes [14,16]. Plasmatocytes represent more than 90% of the hemocyte pool. These cells have been shown in vitro to possess strong adhesive features, enabling them to surround and engulf pathogens, and to produce antimicrobial peptides (AMPs) for the humoral defense [14,15]. Unlike plasmatocytes, crystal cells are not adhesive, but they can express phenoloxidase, the key enzyme in the formation of melanin involved in wound healing and melanization [17]. Lamellocytes are large adhesive cells that are only present in larva or in infected adults, and are involved in melanization and encapsulation [15].

## 3. Insect Lectins

Insects are the largest and most diverse group of animals, and more and more insect lectins are being discovered. Lectin classification is important to cope with the diversity of these proteins. Insect lectins can be grouped according to the animal classification system, which encompasses 16 families of lectins, each with a characteristic carbohydrate-recognition domain (CRD) [18].

In insects, most of the identified endogenous lectins belong to the C-type lectin (CTL) family. Canonical CTLs bind sugars through their CRD, and this interaction is dependent on Ca^2+^, hence the name “C-type lectins”. The CRD motif of CTLs is versatile, resulting in broad range of carbohydrate-binding interactions. For example, the Glu-Pro-Asn (EPN) motif in the CRD binds mannose, N-acetylglucosamine, L-fucose, and glucose, while galactose and N-acetylgalactosamine are recognized by the Gln-Pro-Asp (QPD) motif [19,20]. Many other motifs have been identified in insects, such as QPS, QPN, APD, and MPP, among others [21], but their carbohydrate-binding activities need to be confirmed. According to their complexity, CTLs can further be classified into subfamilies such as collectins (collagen-containing C-type lectins), endocytic receptors, selectins, etc. [22]. Based on sequence homology, proteins with a CTL domain have been identified in at least 12 insects belonging to different orders, including model organisms such as *Drosophila melanogaster*, *Bombyx mori*, *Manduca sexta*, *Tribolium castaneum* and *Nilaparvata lugens* [9,21]. Expression of some of these putative lectins was verified by quantitative real-time PCR [21]. In each of these insect genomes, about 7–40 putative CTLs have been identified and most contain a signal peptide, indicating these proteins are probably secreted extracellularly [9]. The majority of these CTLs have a single CRD, but *M. sexta*, *Helicoverpa armigera* and *Spodoptera litura* possess lectins with a dual-CRD structure (also named the immulectin family). The CTL domain can be linked to other functional domains (CTL-X) such as an epidermal-growth-factor-like domain (EGF) or a chitin-binding domain (CBM), which greatly increases the functional diversity among CTLs [9]. Being the largest lectin family in insects, CTLs are involved in a broad range of processes, especially the immune responses (Table 1).

Malectin and calnexin/calreticulin are protein chaperones located in the ER (endoplasmic reticulum). During translation, an N-glycan precursor (Glc3Man9GlcNAc2) is attached to the newly synthesized polypeptide. The processing of the precursor glycan by glucosidases yields bi-, mono-, and non-glucosylated N-glycans, which creates signals for glycoprotein folding and quality control mediated by the chaperone lectins. Malectin binds to Glc2-N-glycans, whereas calnexin/calreticulin binds to Glc1-N-glycans [6,23,24]. Malectins in the invertebrate scallop *Chlamys farreri* and big-belly seahorse *Hippocampus abdominalis* are regulated by pathogen infection [25,26], suggesting their participation in immunity. Orthologs of malectins have been identified in *D. melanogaster* and *A. aegypti*, but have not been studied yet [27,28]. Calnexin/calreticulin chaperones have been identified in *B. mori* [29,30] and *D. melanogaster* [31,32]. In *Drosophila*, calnexin was reported to be related to neuron functions and sodium channel regulation [31,32].

F-type lectins (FTL) preferentially bind to fucose through a carbohydrate-binding domain composed of the HX(26)RXDX(4)R/K sequence motif [33,34]. The first FTL identified in insects was the lectin encoded by the *Drosophila furrowed* gene, and the furrowed protein is associated with a CTL domain and Sushi repeats [33,35,36]. *Drosophila* furrowed participates in planar cell polarity signaling and is crucial for cell adhesion [37]. The F-type lectin domain is also predicted in *Anopheles gambiae*, but its function has not been verified yet [35,36].

Chitinase-like proteins (CLPs) gained their name due to their chitin-binding ability. In contrast to chitinases, these proteins lack the enzymatic activity to digest chitin due to the absence of essential catalytic residues in the consensus motif [38]. In *Drosophila*, the most notable CLPs are the imaginal disc growth factors (IDGFs), composed of six glycoproteins which participate in cellular functions like proliferation, mobility, and immune recognition [38,39]. Sequences encoding CLPs have been predicted in at least in 10 insects including model insects like the red flour beetle, *T. castaneum*, *N. lugens*, and mosquito, *A. gambiae*; sequences encoding CLPs were predicted, but since the homology search is based on a motif of catalytic residues, some of these CLPs identified are actually true chitinases [40,41,42,43], which are normally not considered to be lectins [44,45].

L-type lectins are soluble ER luminal compounds which contain a CRD similar to those of leguminous plant lectins such as concanavalin A (Con A), and some L-type lectins are responsible for glycoprotein sorting and trafficking [20,46]. *Drosophila* has a homolog of ER–Golgi intermediate compartment 53 (ERGIC-53), a human L-type lectin responsible for cargo transport of glycoproteins [47,48], which may be related to the adhesion protein talin [49]. *B. mori* also has an ERGIC-53 homolog which responds to insecticide treatment [50]. The L-type lectin LvLTLC1 was reported to be upregulated after pathogen stimuli in shrimp [46], but this was not reported in insects.

Galectins or S-type lectins contain a CRD that specifically binds to β-galactosides [51], although other carbohydrate ligands have also been reported. For example, the galectin Agalectin from *A. gambiae* caused agglutination that was inhibited by gangliosides, sulfated polysaccharides, and sialic acid-containing glycans [52,53]. Galectins in human can be further classified into three major groups: prototypical galectins, chimeric lectins, and tandem-repeat galectins, according to their CRD organization [54]. Many animal lectins are glycosylated, but the galectin family seems to be an exception [55,56]. Galectins have been reported in a few insects, including *D. melanogaster*, *A. gambiae, A. aegypti*, and the sand fly *Phlebotomus papatasi* [57,58,59,60,61]. Galectins expressed in the insect gut have been shown to participate in the neutralization of bacterial toxins [57,58].

I-type lectins belong to the immunoglobulin gene superfamily (IgSF). Hemolins, the well-studied I-type lectins of *D. melanogaster*, *S. exigua*, and *M. sexta*, recognize lipopolysaccharides, and their expression was shown to be induced after bacterial infection [62]. Further studies suggest that hemolin facilitates phagocytosis of bacteria and encapsulation of synthetic beads [62,63,64].

R-type lectins have a CRD similar to ricin, the toxic plant lectin from castor bean. Most R-type CRDs are ligated to other functional domains, including the CTL domain (mannose receptor family), pore-forming domain, and GalNAc-transferase domain. In the genome of *D. melanogaster*, 14 GalNAc-transferases have been identified containing R-type CRDs at their carboxy terminals. A QxW repeat in the CRD was supposed to be an important motif for carbohydrate binding [65,66].

Other lectin families common in animals, such as P-type and X-type lectins, are seldom identified in invertebrates [18] although previous searches in insect genome sequences predicted their existence [20].

**Table 1:** Overview of insect lectins.

Lectin Families	Insect Species	Gene/Protein ^a^	Lectin Functions	Experiment Verification		Predicted by GO/Homology	References
				RNA ^b^	Protein ^c^		
CTL	*Aedes aegypti*	AaeCTLs; CTL-20; mosGCTL-7	Pathogen recognition; interacts with phosphatase; reduces exogenous toxin toxicity	+	+		[9,67,68,69]
	*Tribolium castaneum*	TcCTL6, TcCTL3	Responds to pathogen infection; regulates AMP expression		+		[70,71]
	*Spodoptera litura*	*SliCTLs*	Responds to pathogen infection	+			[21]
	*Mythimna separata*	EPL	Promotes encapsulation	+			[72]
	*Ostrinia furnacalis*	OfCTLs, OfIMLs		+			[73]
	*Spodoptera exigua*	Se-LLs, Se-BLLs	Responds to virus infection	+			[74]
	*Thitarodes xiaojinensis*	*CTL-S*, *CTL-X*, *IMLs*	Responds to pathogen infection	+			[75]
	*Helicoverpa armigera*	Ha-lectin, HaCTL	Regulates ecdysone and juvenile hormone signaling; regulates AMP expression; promotes phagocytosis		+		[76]
	*Drosophila melanogaster*	Slf, DL2-3	Organizes the cuticle layers; enhances encapsulation		+		[77,78]
	*Antheraea pernyi*	Ap-CT	Binds PAMPs; activates PO		+		
	*Bombyx mori*	BmIML, BmMBP, CTL-S3, BmEL-1, 2, 3	Recognizes PAMPs; activates PO; promotes melanization;		+		
	*Hyphantria cunea*	*Hdd15*		+			
	*Periplaneta americana*	LPS-BP	Responds to *E. coli*		+		
	*Heliothis virescens*	MBL			+		Reviewed by [9]
	*Manduca sexta*	MsIML-1, 2, 3, 4	Responds to pathogens; binds PAMPs; activates PO; enhances encapsulation		+		
	*Anopheles gambiae*	AgamCTLs	Responds to pathogens				
	*Nilaparvata lugens*		n.d.				
	*Plutella xylostella*		n.d.				
	*Apis mellifera*		n.d.				
	*Acyrthosiphon pisum*		n.d.				
Chitinase like	*Acyrthosiphon pisum*	*AcypiCht1* (IDGF homologue)	Expresses in bacteriocyte and midgut	+			[41]
	*Anopheles gambiae*	*AgIDGF2*, *AgIDGF*4	Expresses in different developmental stages and tissues	+			[79]
	*Bombyx mori*	BmIDGF	Expresses in eggs, hemocytes, fat body, and silk gland		+		[80,81]
	*Drosophila melanogaster*	IDGF1-6	Participates in would healing and wing development	+	+		[38,39,82]
	*Nilaparvata lugens*	*NlIDGF*	Expresses in female reproductive organs and fat body	+			[42]
	*Tribolium castaneum*	*TcIDGF2, 4*	Acts in adult eclosion	+			[83]
	*Plutella xylostella*	*PxIDGF*	n.d.			+	[84]
	*Manduca sexta*	*MsIDGF1*	n.d.			+	[85]
	*Bemisia tabaci*	*BtIDGF1-3*	Highly abundant in adults	+			[86]
Galectin	*Drosophila melanogaster*	Dmgal	Expresses in hemocytes and in different developmental stages		+		[59,87]
	*Phlebotomus papatasi*	PpGalec	Strong expression in adult female; binds pathogen				[61]
	*Anopheles gambiae*	*Agalectin*, *GALE6-8*	Expresses in salivary gland; Responds to viral infection	+	+		[52,88]
	*Bombyx mori*	BmGalectin-4	Responds to bacteria in fertilized eggs; binds bacteria		+		[89]
	*Aedes aegypti*	galectin-6, galectin-14	Reduces exogenous toxin toxicity		+		[57,58]
	*Anopheles darlingi*		n.d.				
	*Anopheles stephensi*		n.d.				
	*Culex quinquefasciatus*		n.d.				
	*Drosophila ananassae*		n.d.				
	*Drosophila mojavensis*		n.d.				
	*Drosophila pseudoobscura*		n.d.				
	*Drosophila virilis*		n.d.			+	Predicted by [87]
	*Drosophila willistoni*		n.d.				
	*Drosophila yakuba*		n.d.				
	*Glossina morsitans*		n.d.				
	*Malus domestica*		n.d.				
malectin	*Aedes aegypti*		n.d.			+	[27,28]
	*Drosophila melanogaster*		n.d.			+	
Calnexin/calreticulin	*Bombyx mori*	Calr/Canx; BmCNX	Responds to ER stress	+	+		[30,90]
	*Drosophila melanogaster*	Cnx	Regulates the function of sodium channel paralytic		+		[32]
F-type lectin	*Drosophila melanogaster*	Furrowed	Functions in planar cell polarity		+		[37]
	*Anopheles gambiae*		n.d.				Reviewed by [36]
I-type (immuno-globulin fold)	*Drosophila melanogaster*	hemolin	n.d.		+		Reviewed by [91]
	*Manduca sexta*	HEM	Recognizes PAMPs; promotes nodulation, hemocyte aggregation, and phagocytosis				[63]
	*Spodoptera exigua*	*SeHem*	Acts as opsonin; regulates phagocytic activities and encapsulation	+			[62]
	*Plodia interpunctella*	PiHem	Function related to gut bacteria	+			[92]
	*Bombyx mori*	Hemolin	n.d.			+	[93]
	*Actias selene*	As-HEM	Mediates immune response		+		[94]
	*Antheraea pernyi*	Hemolin	Regulates innate immunity		+		[95]
L-type	*Drosophila melanogaster*	ERGIC-53 homolog	n.d.				[48], reviewed by [96]
	*Bombyx mori*	*ERGIC-53*	Responds to ER stress	+			[50]
R-type (ricin B type)	*Drosophila melanogaster*	lectin domain of GalNAc Transferase	Binds glycopeptides		+		[97], reviewed by [65]

^a^ some publications have predicted lectins but did not assign names for these lectins; therefore, there are some blanks in the table. ^b^ RNA verification studies included RT-qPCR, dsRNA silencing, and transcriptome analysis. ^c^ Protein verification included immunoblotting, recombinant protein production, etc.

## 4. Endogenous Insect Lectins as Immune Modulators

### 4.1. Pathogen Recognition

Before hemocytes can activate the immune response, the pathogen or immune target must be recognized. During pathogen invasion, pathogen-associated molecular patterns (PAMPs) such as bacterial peptidoglycan or fungal β-1,3-glucan are recognized by specialized proteins called pattern-recognition receptors (PRRs) [98]. The Gram-negative binding proteins (GNBPs), and peptidoglycan-recognition proteins (PGRPs) are the two major PRR families. GNBPs mainly recognize fungal and Gram-negative bacterial PAMPs, while PGRPs mainly respond to Gram-positive bacteria [98]. Since many PAMPs are carbohydrate structures, lectins constitute important parts of the membrane-bound or extracellular PRRs of hosts.

Lectins have been reported to bind and aggregate pathogens such as bacteria because of their recognition of carbohydrate structures. CTLs of *H. armigera* and *M. sexta* were shown to bind various PAMPs, such as lipopolysaccharide (LPS), fungal glucan, and peptidoglycan, to activate the humoral and cellular immune defenses [9,76,99,100]. In *Drosophila*, CTLs such as DL2 and DL3 can either be secreted or bound to the plasma membranes of hemocytes, and they were shown to bind some Gram-negative bacteria and agglutinate them [15]. While many insect PRRs belong to the C-type lectin family, lectins from other families can also function as PRRs. For example, galectins have been reported to recognize and bind pathogen surface glycans [53]. The silkworm *B. mori* possesses a dual-CRD galectin which can bind a series of PAMPs, such as LPS, LTA (lipoteichoic acid), peptidoglycan, and laminarin, and was shown to agglutinate *E. coli*, *Staphylococcus aureus*, and *Bacillus subtilis* [89,101].

### 4.2. Lectin-Induced Cellular Immunity

#### 4.2.1. Phagocytosis

Many hemocytes can engulf invading pathogens as well as dead cells or other entities in a process called phagocytosis [13,102]. Upstream events of phagocytosis include the recognition of the targets by the PRRs, which activates downstream events including receptor cross-linking, membrane remodeling, phagosome formation, and maturation, and finally phagosome fusion with the endosomes and lysosomes to kill the pathogens via the acidic environment, AMPs, digestive enzymes, etc. [103]. To increase the efficiency of phagocytosis, hemocytes sometimes rely on opsonins, molecules that can coat and aggregate pathogens such as bacteria and viruses to limit their mobility and promote recognition [103]. Lectins have been proven to stimulate phagocytosis by acting as PRRs to detect pathogens or as opsonins to coat the invaders. For example, rHa, a CTL lectin obtained from *H. armigera*, has two CRDs which are both required for lectin agglutination of rabbit erythrocytes, but any single domain is sufficient for aggregation of Gram-negative bacteria, Gram-positive bacteria, and fungi. Injection of rHa lectin together with *Bacillus thuringiensis* bacteria in insects efficiently decreased the *B. thuringiensis* number in vivo, and hemocytes of *H. armigera* engulfed more *B. thuringiensis* in the presence of rHa lectin [104]. CTL-mediated phagocytosis has also been observed in mammalians and shrimps [105,106]. Besides the CTLs, the I-type lectin hemolin SeHem from *S. exigua* also helped the host cells to eliminate bacteria by enhancing phagocytosis [62]. Galectins from crustaceans have been reported to enhance host phagocytosis [107] but there are no such reports for insect galectins.

#### 4.2.2. Encapsulation

When the invading targets are too large, such as parasitoids or nematodes, a group of hemocytes is recruited to surround the target, forming a capsule-like structure in a process termed encapsulation. In *Drosophila*, lamellocyte precursor cells are activated upon infection with parasitic wasp eggs and will differentiate into mature forms [16,108]. These cells are recruited to the site of infection, attach to the surface of the parasites, and undergo morphological changes to spread around the parasitoids [16]. The process in which the lamellocytes are flattened is called cell spreading and relies on phosphatase/kinase-mediated cytoskeleton rearrangement and activation of adhesion proteins [109,110,111]. Cell spreading is a very fast reaction; a mere 20 min after stimulation, most *Drosophila* S2 cells had already entered this spreading state [112]. The spread cells cover the parasite to form the capsule. Stabilization of the capsule depends first on intercellular septate junctions. These ladder-like structures are composed of multiple adhesion proteins such as contactin, neurexin, fibronectin, etc. [113]. Second, melanization follows to strengthen the capsule and to kill the parasites. Melanization is a process in which phenols are oxidized to quinones that can be polymerized to form melanin [114]. The deposition of melanin will darken the capsule [115]. Encapsulated targets are restricted in their movement and are finally killed directly by melanization-derived toxic components such as quinones, reactive oxygen intermediates, and AMPs [115], or indirectly by nutrient deprivation [116].

Insect lectins have been shown to be involved in both encapsulation and melanization. One common method used to study encapsulation in vitro is the use of synthetic beads incubated with isolated hemocytes. Synthetic beads such as agarose or Sephadex can attract hemocytes which form capsules around the beads that can be easily observed under a microscope [109]. Coating of these beads with stimulating proteins can accelerate and increase encapsulation [78]. For example, recombinantly produced *Drosophila* CTLs DL2 and DL3 were coated onto Ni-NTA agarose beads which can attract hemocyte attachment. These hemocytes aggregated to the bead surface to form capsules and became darker colored after longer incubations, and this process was blocked by antibodies targeted against the recombinant proteins [78]. Besides the in vitro test, injecting the coated beads into an insect hemocoel also validated the hypothesis. In *H. armigera*, a CTL, HaCTL3, was coated onto Sephadex A-25 beads and injected into the *H. armigera* larval hemocoel. After 12 h, the beads were found to be extensively encapsulated and melanized [99]. Besides CTLs, the I-type lectin SeHem was also reported to coat nonself targets for encapsulation [62]. While many lectins have been reported to participate in encapsulation and melanization [9], it is not very clear which receptors on the plasma membrane are responsible for the effect. One possible explanation is through interaction with integrins. Evidence suggests that silencing of β-integrin, a hemocyte membrane protein participating in cell–cell adhesion and signal transduction, can effectively decrease the encapsulation of beads [99]. In addition, CTL-mediated melanization is suggested to be specific. In one in vitro test, the immune lectin MsIML from *M. sexta* was shown to be able to activate a protease cascade required for phenoloxidase activation, which only happens when this lectin binds to LPS. Phenoloxidase has been proven to be a key enzyme for melanization [114,117].

### 4.3. Lectin-Induced AMP Expression

Besides the cellular response, the insect host can secrete a series of extracellular effector molecules that can kill foreign invaders. Among these effectors, AMPs are the major participants [118]. AMPs are positively charged small peptides consisting of 15–45 amino acids, which can bind to the normally negatively charged surface of microbes and lead to membrane rupture and cell lysis [119]. The healthy host cells are protected from AMP damage mainly by the cholesterol-rich plasma membrane which makes healthy cells positively charged to repulse cationic AMP attachment [119]. However, when host cells are not healthy, they can be attacked by the AMPs. In a study of *Drosophila* tumor genesis, tumor cells tended to have a negatively charged cell surface due to the phosphatidylserine turning inside out, allowing the AMP defensin to locate and attack these cells to limit the tumor growth [120].

Classification of AMPs can vary based on different criteria, such as the type of the target microbe (antifungal or anti-Gram-positive/negative-bacterial AMPs) or based on the pathway by which they are activated (such as Toll-regulated or Imd-regulated AMPs) [118]. However, neither classification system can perfectly group different AMPs. While AMPs like drosomycin (Drs) are highly specific against fungal infections, other AMPs have a broader pathogen specificity. For example, metchnikowin (Mtk) can target all three groups of pathogens mentioned above. In addition, AMP regulation can also be complex; for example, while Drs is regulated by the Toll pathway, many others, such as defensin (Def), are coregulated by both pathways [118,119].

The insect fat body around the body cavity is the major tissue secreting AMPs [121]. When stimulated by a pathogen, the AMP titers in the hemolymph can drastically increase within 30 min and the concentration can reach up to 300 µM (reviewed by [15]). In addition to the fat body cells, hemocytes can also produce AMPs. For example, isolated hemocytes from the blue blowfly, *Calliphora vicina*, showed the same ability to produce AMPs such as defensin, cecropin, diptericins, and proline-rich peptides [111]. *Drosophila* hemocyte-like S2 cells have also been shown to produce all kinds of AMPs upon stimulation by *E. coli* or other protein stimuli [112,122].

Insect lectin-mediated pathogen recognition can trigger the production of AMPs. Insect lectins are commonly coregulated with AMPs [123], but a recent study gave more direct evidence that the insect lectin can regulate AMPs. After silencing of HaCTL3, a CTL from *H. armigera* participating in larval development, the fat body expressed far less AMPs than in the control group. HaCTL3 was found to regulate different AMPs, including *lebocin*, *attacin*, *cecropin 1*, *pre-gloverin*, *pre-lebocin*, and *cecropin*, of which the antimicrobial activities were confirmed by in vitro assays. Even more interestingly, the upstream PRRs, including PGRPs, β-1,3-GRPs, and even a CTL4, were also downregulated, suggesting that lectin-regulated AMP production might be initiated by affecting upstream recognition events [76].

Within host immunity, insect lectins and AMPs can have complex interactions. Insect lectins can protect the beneficial host microbiome against the toxic effects of AMPs. For example, silencing of *A. aegypti* C-type lectins (mosGCTLs), which are coregulated with AMP through the Imd pathway, leads to failure of colonization and maintenance of the gut microbial flora [124]. In addition, AMP toxicity significantly decreased when bacteria were pre-coated by mosGCTLs, which blocked AMP deposition on the bacterial surface [124]. Viruses are also reported to use host lectins. The West Nile virus (WNV), a pathogen causing West Nile fever and transmitted by mosquitos, can stimulate expression of an *A. aegypti* C-type lectin, mosGCTL-1, which can strongly interact with a mosquito phosphatase, mosPTP-1. WNV uses mosGCTL-1 to coat its surface and enters cells through interaction with mosPTP-1 [125].

## 5. Plant Lectins as Exogenous Immune Modulators

**Figure 1:**
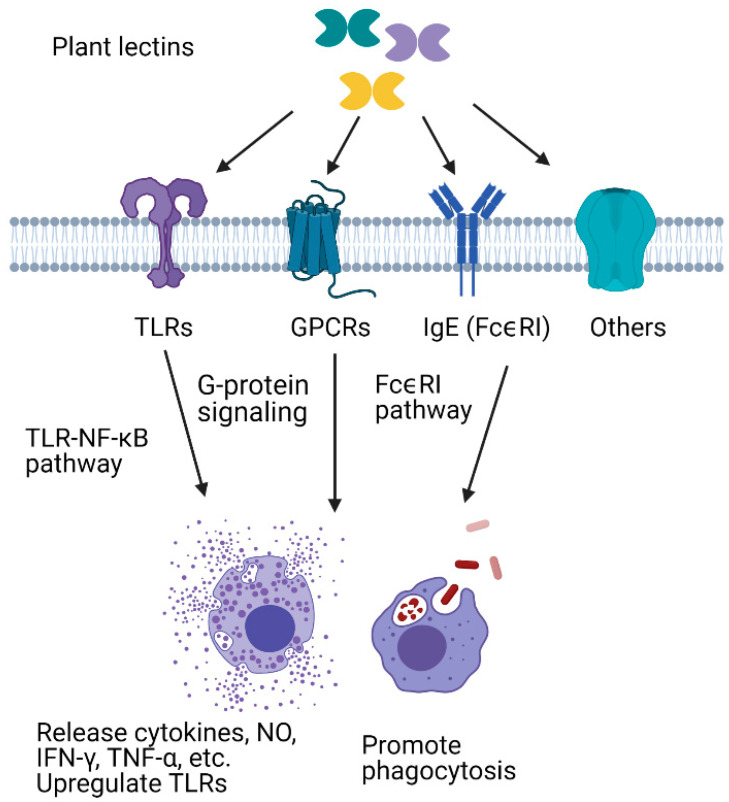
Immunomodulatory effects of plant lectins.

Similarly to the animal lectins, plant lectins have many important biological functions. In addition, many plant lectins possess entomotoxic properties, which has led to interest in these proteins for the development of novel pest control strategies. In addition, in recent years, the immunomodulatory effects of plant lectins have attracted attention for potential medical and pharmaceutical applications. In mammalians, it has been shown that many plant lectins display immunomodulatory activities after interaction with glycan moieties of immune cells (Figure 1).

While the knowledge of the endogenous insect lectins is lagging, the knowledge of the molecular effects of plant lectins might be useful in elucidating the functions and modes of action of insect lectins in the immune system.

### 5.1. Immunomodulatory Effects of Plant Lectins in Mammalian Cells

#### 5.1.1. Plant Lectins Interact with Immune-Related Proteins and Enhance Pathogen Recognition

Upstream events of immunomodulatory plant lectins involve interactions with proteins of mammalian immune cells. ArtinM (also known as KM^+^) is a mannose-specific jacalin-related lectin from *Artocarpus heterophyllus* (jackfruit) seeds. ArtinM was reported to interact with the neutrophile cellular surface receptor CXCR2 through binding to its N-glycan [126], triggering G-protein dependent signaling and tyrosine phosphorylation for downstream effects [127]. Since mannose can block these upstream events, the lectin–carbohydrate interaction seemed to be crucial to the ArtinM immunomodulatory effects. ArtinM was also reported to interact with the neutrophil laminin [128], mast cell IgE receptor (Fcε receptor) [129], and macrophage Toll-like receptor 2 (TLR2) [130].

TLRs, the mammalian orthologs of the *Drosophila* immune receptor Toll, are important PRRs of mammalian cells that can be targeted by plant lectins. In vertebrates, 13 TRLs with different immune functions have been identified [131]. For example, cell-surface TLRs, including TLR1, 2, 4–6, and 10, recognize microbial PAMPs such as lipoproteins, lipids, and other microbial membrane fractions, while the endosomal TLRs, i.e., TLR3, 7–9, have been suggested to recognize the invading nucleotides from viruses and bacteria [131]. Next to ArtinM, plant lectins such as Con A, WGA and PHA-L have been shown to target TLRs and, in addition, to enhance TLR expression [132,133]. Similarly, the Korean mistletoe lectin (KML-C), phytohemagglutinin (PHA) and its isoforms, SBA (soybean agglutinin), PNA (peanut agglutinin), etc., were found to stimulate TLRs [132]. For example, the expression of a series of TLRs (TLR2–9) in murine peritoneal macrophages was enhanced through the JNK pathway after treatment with the plant lectin Con A [132,134,135]. These TLRs are heterodimerized, which clearly improved host-cell sensitivity to PAMP stimuli such as LPS [132,135]. In addition, Con A induced humoral immunity through activation of the TLR-NF-κB pathway [131]. Some chimeric lectins have been shown to stimulate complex cellular responses. For example, the European mistletoe lectin (ML) is composed of two chains, A and B. The A chain is responsible for inactivation of the ribosome, causing cell toxicity. The B chain is able to bind the cellular surface through glycan binding, and the B chain alone is sufficient to stimulate the immune response [136].

#### 5.1.2. Plant Lectins Enhance Phagocytosis

Plant lectins can promote cellular responses. pCramoll is the seed lectin from *Cratylia mollis*, and co-incubation with mice peritoneal exudate cells increased the cellular phagocytosis of the pathogen *Staphylococcus aureus* by 27% [137]. In addition, pCramoll also increased the mouse macrophages’ ability to phagocytose the pathogen *Cryptococcus gattii* [138]. In the presence of garlic lectin (ASA I and ASA II), the macrophages increased their phagocytosis of yeast by about 80% [139]. Neutrophiles are another important component of the mammalian cellular defense. ArtinM was shown to stimulate immune neutrophil phagocytosis. ArtinM-treated human neutrophils displayed increased phagocytosis of both fluorescently labeled synthetic zymosan and the pathogen *Listeria monocytogenes*, which was accompanied by an increased lysosome volume [127]. Many more plant lectins have been reported to activate neutrophils and lead to cellular defense (reviewed by [140]).

#### 5.1.3. Plant Lectins Promote the Release of Cytokines and Other Effectors

Plant lectins can induce the expression of humoral effectors against tumors or microbial infections [136,141,142,143]. Activated macrophages can produce cytokine signal molecules. Cytokines are a group of small molecules that participate in intercellular communication. Once cytokines are bound to the receptors of recipient cells, a series of cellular events, depending on the cell type, will be initiated. Immunomodulatory cytokines constitute a large family, including interferons, interleukins, and tumor necrosis factors (TNF) [144,145]. For a long time, plant lectins have been linked to cytokine release. The lectins stimulate cytokine production in different types of immune-related mammalian cells, such as murine macrophages, spleen cells, dendritic cells, mouse spleen cells, and human dendritic cells, neutrophils, peripheral blood mononuclear cells, etc. [136,142,143].

In a mouse model, Con A treatment upregulated the expression of inflammatory cytokines, including IL-2, 6, 10, 17A, IFN-γ, and TNF-α, up to 100-fold [134]. These increased cytokine levels subsequently activate the release of cytotoxic chemicals, for example the oxidative stress marker NO (nitric oxide), which is toxic towards infectious organisms [134,146]. IL12 upregulation, mediated by ArtinM, was required to protect the mouse host against infection by *Paracoccidioides brasiliensis* [130]. ArtinM-activated neutrophils produced twice the IL-8 levels compared to the control cells, which was accompanied by a 3-fold increase in superoxide release [127]. The increased ROS production, normally triggered by pathogen infection, after treatment with the plant lectin pCramoll was shown to facilitate the elimination of pathogens such as *Cryptococcus gattii* [147].

### 5.2. Plant Lectins as a Tool to Study Insect Immune Responses

In insects, the modulation of the immune responses by exogenously applied plant lectins is not well elucidated, and only a few studies have indicated their immunomodulatory effects. For example, feeding *D. melanogaster* with phytohemagglutinin (PHA), a lectin from the seeds of the red kidney bean *P. vulgaris*, induced the expression of AMPs such as attactin-A, defensin, and drosomycin, along with a drastic upregulation of the phagocytosis proteins Draper and integrin βν [148]. In addition, a transcriptome analysis in *D. melanogaster* showed that feeding on a WGA-mixed diet increased the transcription levels of some immune-related peptidases and downregulated some lysozymes, similarly to the effects observed after viral infection [149].

While the plant-lectin-induced insect immune responses are far from being clear, plant lectins can still be useful tools to investigate insect innate immunity. First of all, in mammalian systems, plant lectins have been analyzed in more detail for their immunomodulatory effects, especially regarding receptors on the cell membrane and in the cytosol, in both the cellular and humoral immune responses [136,141,142,143]. In addition, the signaling pathways behind these responses have been extensively studied in vertebrate models [131]. This knowledge can inspire the identification of orthologs in insects. Second, a huge number of plant lectins have been identified and their basic characteristics are elucidated [150], offering a ready-to-use option to study insect immune responses. Many plant lectins participate in plant host immunity by recognizing different pathogen derivatives such as bacterial and fungal exopolysaccharides (reviewed by [151]) and metabolites [152]. Some interesting features of plant lectins can help researchers to generate a working hypothesis related to the functions of insect lectins in the immune response. For example, the lectin domain of *Arabidopsis* LecRKs (lectin receptor kinase) lacks the critical motif for carbohydrate binding; however, LecRK can activate the calcium response, kinase activation, and downstream gene expression, through lectin-domain binding to eATP (extracellular ATP) [153] and eNAD^+^ [154], which are important extracellular signals in immunity of both plants (reviewed by [155]) and animals (reviewed by [156]). A recent study also indicated that carbohydrate binding is not necessary for plant-lectin-induced insect cellular immunity [157]. These findings suggest that immune functions of lectins can be achieved independent of carbohydrate-binding ability, which is rarely reported in insects. Third, elucidating the immunomodulatory effects of plant lectins in insects can help to understand the naturally occurring plant–insect interactions, which may be useful in protecting beneficial insects and in the development of new pest control strategies.

## Data Availability

No new data provided.
